# Musical hallucinations: review of treatment effects

**DOI:** 10.3389/fpsyg.2015.00814

**Published:** 2015-06-16

**Authors:** Jan A. F. Coebergh, R. F. Lauw, R. Bots, I. E. C. Sommer, J. D. Blom

**Affiliations:** ^1^Department of Neurology, Haga HospitalThe Hague, Netherlands; ^2^Department of Neurology, Ashford/St. Peter's HospitalChertsey, UK; ^3^Department of Neurology, St. George's HospitalLondon, UK; ^4^Parnassia Psychiatric InstituteThe Hague, Netherlands; ^5^‘S Heeren LooNoordwijk, Netherlands; ^6^Department of Psychiatry, University Medical Center UtrechtUtrecht, Netherlands; ^7^Brain Centre Rudolf MagnusUtrecht, Netherlands; ^8^Department of Psychiatry, University of GroningenGroningen, Netherlands

**Keywords:** Auditory Charles Bonnet syndrome, auditory hallucination, brain lesion, epilepsy, intoxication/pharmacology, psychiatric disorder

## Abstract

**Background:** Despite an increased scientific interest in musical hallucinations over the past 25 years, treatment protocols are still lacking. This may well be due to the fact that musical hallucinations have multiple causes, and that published cases are relatively rare.

**Objective:** To review the effects of published treatment methods for musical hallucinations.

**Methods:** A literature search yielded 175 articles discussing a total number of 516 cases, of which 147 articles discussed treatment in 276 individuals. We analyzed the treatment results in relation to the etiological factor considered responsible for the mediation of the musical hallucinations, i.e., idiopathic/hypoacusis, psychiatric disorder, brain lesion, and other pathology, epilepsy or intoxication/pharmacology.

**Results:** Musical hallucinations can disappear without intervention. When hallucinations are bearable, patients can be reassured without any other treatment. However, in other patients musical hallucinations are so disturbing that treatment is indicated. Distinct etiological groups appear to respond differently to treatment. In the hypoacusis group, treating the hearing impairment can yield significant improvement and coping strategies (e.g., more acoustic stimulation) are frequently helpful. Pharmacological treatment methods can also be successful, with antidepressants being possibly more helpful than antiepileptics (which are still better than antipsychotics). The limited use of acetylcholinesterase inhibitors has looked promising. Musical hallucinations occurring as part of a psychiatric disorder tend to respond well to psychopharmacological treatments targeting the underlying disorder. Musical hallucinations experienced in the context of brain injuries and epilepsy tend to respond well to antiepileptics, but their natural course is often benign, irrespective of any pharmacological treatment. When intoxication/pharmacology is the main etiological factor, it is important to stop or switch the causative substance or medication.

**Conclusion:** Treatments for musical hallucinations tend to yield favorable results when they target the main etiological factor of these phenomena. There is a need to establish the natural course of musical hallucinations, their response to non-pharmacological treatments, and their effects on the patient's quality of life. There is also a need to standardize the assessment of treatment responses, and document long-term follow up.

## Introduction

Musical hallucinations (MH) are auditory hallucinations characterized by songs, tunes, melodies, harmonics, rhythms, and/or timbres (Coebergh et al., 2009). The scientific interest in these phenomena has increased significantly over the past 25 years, but so far no evidence-based treatments have been established. The principle reason for this would seem to be that published case series are relatively small, and that the mechanisms responsible for the mediation of MH are probably diverse. Furthermore, case reports tend to over represent positive responses, as negative responses to specific medication are rarely published. We therefore endeavored to identify which treatments have been tried under which circumstances, and what their effect has been upon the MH.

MH are commonly divided into two groups. When they occur without the presence of any associated pathological abnormalities—with the exception of hearing impairment (hypoacusis)—they are called idiopathic MH. When there are associated pathological abnormalities, they are named symptomatic MH (Coebergh et al., 2009). The most relevant etiological factors for MH, as reported in the literature, are brain injuries, epilepsy, psychiatric disorder, and intoxication/pharmacology (Berrios, 1990; Keshavan et al., 1992; Evers and Ellger, 2004; Cope and Baguley, 2009).

Research by Fukunishi et al. (1998a), Schakenraad et al. (2006) and Teunisse and Olde Rikkert (2012) has shown that MH are more common than previously assumed, with prevalence rates of 3.6% among groups of patients referred for audiometric testing. Similar to our own experiences, Ross (1978) and Aziz (2009) found that after the publication of an article on the subject several people wrote to them, and told them that they had adjusted to their MH without seeking any medical attention. However, others show themselves in dire need of medical consultation. MH can indeed be experienced as mild and well-tolerable, but more often they are experienced as severely disabling, leading to impaired quality of life, significant distress, and comorbid anxiety and/or depression. Patients often have difficulty concentrating and falling asleep, and many of them are afraid that they are dementing or otherwise psychiatrically ill (Fukunishi et al., 1998b; Cole et al., 2002; Schakenraad et al., 2006). Occasionally they develop secondary delusions, and accuse others (frequently neighbors) of being responsible for the music. As a result, they may even call the police, move house, or take other drastic measures. Diagnosis and treatment of such cases is paramount, but at this point the lack of treatment protocols and our lack of knowledge about the natural history and prognosis of MH stand in the way of an evidence-based approach.

Various treatment options have been described in case reports and modest case series. Non-pharmacological treatment tends to consist of lifestyle advice, reassurance, and explanation of the nature of MH, optimization of hearing, and behavioral modifications [such as listening to actual music (Coebergh et al., 2009)], but no previous review has recorded the effectiveness of such interventions. Loneliness may be an important factor and increasing social interactions and conversations can be beneficial. All pharmacological treatment of idiopathic MH is off-label. It tends to consist of antiepileptics, antidepressants, antipsychotics or acetylcholinesterase inhibitors, but even when treated for a sufficient amount of time and at adequate plasma levels, MH can be refractory to most of these treatments (Sacks and Blom, 2012). Repetitive transcranial magnetic stimulation (rTMS) may perhaps be a viable option in the near future, but so far it only showed a favorable response in a single medication-resistant patient with post-traumatic damage to the right temporal lobe (Cosentino et al., 2010).

This review attempts to quantify the treatment as responses described in the literature, and to correlate them with the probable etiology of the MH at hand. It should be noted, however, that in the literature we reviewed treatment responses were described and defined in widely varying ways. In some papers there was an extensive description of what exactly changed in e.g., the character, loudness and frequency of the MH, whereas other papers only mentioned that they changed, decreased or stopped or remained unaltered. Duration and onset of treatment response were also reported extremely variably and frequently not mentioned at all.

## Methods

For the purpose of this review, we searched the databases of MEDLINE, psycINFO, and Embase Psychiatry for articles matching the terms Music^*^ hallucin^*^, *Musical hallucination*, and *Treatment*, and added relevant papers from our personal collection with titles such as “Auditory Charles Bonnet syndrome” and “The sound of silence.” We examined all articles published between 1890 and January 2015 that were written in English, Dutch, French or German.

The variables we assessed in each published case report were age, sex, treatment, and results of treatment. When available, documentation of hearing impairment, electroencephalography (EEG), brain imaging, and psychiatric assessment were used to establish our interpretation of the main etiological factor (which was occasionally different from that of the original authors). Five different etiological groups were distinguished consisting of hypoacusis, brain lesions and other pathology, epilepsy, psychiatric disorder, and intoxication/pharmacology. This classification is modeled on Evers and Ellger's categorization of MH in 2004 (Evers and Ellger, 2004). When patients had more than one possible etiology, which turned out to be very common, we chose the one most closely linked to the onset of the MH. For example, if a patient with hypoacusis developed a severe depression, and improved after treatment for depression, then psychiatric disorder was selected as the main cause of the MH. However, when the MH were complicated by depression and did not improve with treatment for depression, than hypoacusis was selected. In two cases no apparent cause was identified (Duncan et al., 1989; Satoh et al., 2007).

All cases were subdivided according to kind of treatment, i.e., non-pharmacological [rTMS, electroconvulsive therapy (ECT), ear-nose-throat intervention, cognitive-behavioral therapy (CBT), coping strategies/explanation], and pharmacological [antidepressants, antipsychotics, antiepileptics, other medication (comprising of benzodiazepines and acetylcholinesterase inhibitors), and withdrawal of medication]. Furthermore, we identified cases where the MH decreased or faded away without any treatment, as well as those where it was documented that the patient desired no treatment whatsoever (which could be for multiple reasons, see the Discussion Section).

When MH appeared soon (i.e., within 1 week) after starting a certain drug or after increasing its dose, and disappeared soon after stopping or reducing the dose, this medication was considered the most likely cause; when patients were occasionally rechallenged and the MH recurred, this was taken as an extra indication that the medication was causative. However, standardized measures of causality such as the WHO-UMC causality assessment system were not applied. At times there were multiple medications, and at other times the exact time course of the relation between the intoxication/pharmacological intervention and MH was unclear.

Epilepsy was deemed the main cause when the MH were phenomenologically linked to a seizure, e.g., as an aura, ictal episode or post-ictal episode, when the patient was treated surgically for an epileptic focus or when epileptiform EEG abnormalities disappeared which had been present prior to treatment.

We scored treatment effects in three groups, i.e., “no effect” (0), “patient experiences a clinically meaningful improvement, but the hallucinations remain” (1), and “complete disappearance of the MH” (2). Improvement was deemed meaningful whenever a report implied that the patient had experienced an improvement in quality of life; irrespective of whether the MH were experienced as quieter, less frequent, or not interfering as much with sleep or daily activities, or whether there was increased acceptance/insight or improved mood/anxiety. Two articles (Bergman et al., 2014; Islam et al., 2014) used the Yale-Brown Obsessive Compulsive Scale (Y-BOCS). At times it was difficult to rate the exact treatment effects; e.g., Warner and Aziz (Warner and Aziz, 2005) briefly described 30 patients, mostly elderly people suffering from hearing loss, and stated that 90% were treated (among which 23 with antipsychotics). The majority lost the MH completely, and the remainder reported a reduction in intensity and frequency of the MH. We chose to conservatively document this as a single patient with hearing loss treated with antipsychotics, with a response of 1. Whenever patients had first tried a certain class of medication without improvement (0), followed by a positive response to another class (1/2), both outcomes were scored.

Author JAFC rated the treatment responses in each report. He is a consultant neurologist who has 9 years of experience with neurological, psychiatric, neurophysiological, and radiological diagnosis, and who personally assessed more than 30 patients experiencing MH. The data were analyzed (and mean treatment response calculated) in Microsoft Excel for Mac 2011.

## Results

A total number of 175 articles were identified that discussed 516 new patients with MH or related symptoms. This collection of articles included various modest case series [(Fenton and McRae, 1989; Mahendran, 2007), one which described 44 patients with MH in association with alcohol hallucinosis (Scott, 1975), and one which described 55 patients diagnosed with obsessive-compulsive disorder (OCD) and/or schizophrenia spectrum disorder where the question was asked whether the patient had *ever* heard music that did not have an external origin (other than humming, thinking of music and simple recollection of music) (Hermesh et al., 2004)].

Among those 175 articles we found 161 that discussed the treatment results of MH or related symptoms (including some which only discussed whether treatment was desired). No more than 28 of those were published before 1990. We took this as confirmation of the above-mentioned increase in the scientific interest in MH over the past 25 years rather than as proof of any increase in their prevalence. We then excluded all reports of musical illusions, pseudohallucinations, palinacusis, hypnagogic or hypnopompic hallucinations, and obsessions (i.e., “earworms”); only rarely did our interpretation of obsession vs. hallucination (i.e., “earworm” vs. MH) differ from that of the original authors (e.g., Islam et al., 2014). A total number of 276 cases in 147 articles met these inclusion criteria (Colman, 1894; Penfield and Erickson, 1941; David et al., 1944; Mulder and Daly, 1952; Rozanski and Rosen, 1952; Arieff and Brooks, 1958; Rennie, 1964; Ross et al., 1975; Scott, 1975, 1979; Schiffter and Straschill, 1977; Miller and Crosby, 1979; Raghuram et al., 1980; Wieser, 1980; Gilchrist and Kalucy, 1983; Hammeke et al., 1983; Mackworth-Young, 1983; Aizenberg et al., 1986, 1987, 1991; Jonas, 1986; Cambier et al., 1987; Lanska et al., 1987; Patel et al., 1987; Keshavan et al., 1988, 1992; Duncan et al., 1989; Fenton and McRae, 1989; Wengel et al., 1989; Berrios, 1990; McLoughlin, 1990; Donnet and Régis, 1991; Fisman, 1991; Nevins, 1991; Podoll et al., 1991; Shapiro et al., 1991; Vallada and Gentil, 1991; Wagner and Gertz, 1991; Freeland and O'Reilly, 1992; Klostermann et al., 1992; Paquier et al., 1992; Erkwoh et al., 1993; Feehan and Birchwood, 1993; Fénelon et al., 1993; Gilbert, 1993; Inzelberg et al., 1993; Isaacson et al., 1993; Couper, 1994; Hosty, 1994; Murata et al., 1994; Terao, 1995; Wodarz et al., 1995; Baurier and Tuca, 1996; Gertz et al., 1996; Stephane and Hsu, 1996; Douen and Bourque, 1997; Marneros et al., 1997; Thorpe, 1997; Clark, 1998; Fernandez and Crowther, 1998; Fukunishi et al., 1998b, 1999; Terao and Tani, 1998; Baba and Hamada, 1999; Terao and Matsunaga, 1999; Zungu-Dirwayi et al., 1999; David and Fernandez, 2000; Gomibuchi et al., 2000; Iijima et al., 2000; Keeley et al., 2000; Schielke et al., 2000; Augustin et al., 2001; Cerrato et al., 2001; Roberts et al., 2001; Tanriverdi et al., 2001; Ali, 2002; Curtin and Remund, 2002; Evers et al., 2002; Izumi et al., 2002; Assal, 2003; Baba et al., 2003; Brusa et al., 2003; Ginsberg, 2003; Matsui et al., 2003; Moore, 2003; Shinosaki et al., 2003; Stricker and Winger, 2003; Agrawal and Sherman, 2004; Aziz et al., 2004; Fischer et al., 2004; Hageman et al., 2004; Kobayashi et al., 2004; Wilkinson and Schuller, 2004; Davies and Quinn, 2005; Wang, 2005; Biran and Steiner, 2006; Janakiraman et al., 2006; Gentile et al., 2007; Gilhuis et al., 2007; Mahendran, 2007; Satoh et al., 2007; Tomar and Cheung, 2007; Ukai et al., 2007; Allen, 2008; Holroyd and Sabeen, 2008; Mocellin et al., 2008; Umene et al., 2008; Williams et al., 2008; Auffarth and Kropp, 2009; Coebergh et al., 2009; Engmann and Reuter, 2009; Ergün et al., 2009; Rentrop et al., 2009; Strauss and Gertz, 2009; Tuerlings et al., 2009; Cosentino et al., 2010; Gondim et al., 2010; Isolan et al., 2010; Kanemura et al., 2010; Mittal and Giron, 2010; Padala et al., 2010; Shoyama et al., 2010; Wong and Bhalerao, 2010; Bleich-Cohen et al., 2011; Huntley et al., 2011; Bhatt and de Carpentier, 2012; Calabrò et al., 2012; Johns and Zuromskis, 2012; Ozsarac et al., 2012; Serrador-García et al., 2012; Simões et al., 2012; Teunisse and Olde Rikkert, 2012; Dinges et al., 2013; Giermanski et al., 2013; Vitorovic and Biller, 2013; Abdel-Aziz and Pomeroy, 2014; Aizawa et al., 2014; Bergman et al., 2014; Colon-Rivera and Oldham, 2014; Futamura et al., 2014; Husain et al., 2014; Islam et al., 2014; Kataoka and Ueno, 2014; Kumar et al., 2014; Mansoor and Ganzini, 2014; Woo et al., 2014; Blom et al., 2015; Joe et al., 2015).

### Clinical features

The clinical features of those 276 cases are shown in Table 1, and the main etiology in Table 2. Among the cases where sex was stated, women made up 68.2 % of the population. The patients' average age was 63.8 years (range 18–94 years). *One hundred* and sixty-six of the patients (60.4%) had a history of hearing impairment, most of them chronic. The level of hearing impairment—even whether a hearing aid was required and/or used, or which frequencies and/or ear were affected—was often not specified. There was a concomitant psychiatric disorder in 99 (36.2%) of the patients; among them, 69.7% suffered from mood disorder. Criteria for diagnosing psychiatric disorder were frequently not recorded, and standardized diagnostic methods were rarely used.

**Table 1:** **Clinical features of 276 patient with musical hallucinations**.

**Sex (*N* = 261)**	**Percentage**
Female (*N* = 178)	68.2%
Male (*N* = 83)	31.8%
Age (*N* = 238)	
Mean	63.8 year (18–94)
Hearing impairment (*N* = 166)	60.4%
Psychiatric disorder (*N* = 99)	36.2%
Depression (*N* = 52)	
Other mood disorder (*N* = 2)	
Schizophrenia spectrum disorder (*N* = 18)	
OCD (*N* = 4)	
Dementia (*N* = 6)	
Anxiety (with or without comorbid depression) (*N* = 15)	
Other (*N* = 2)	

**Table 2:** **Main etiology**.

**Main etiology**	***N***	**Percentage (%)**
Hypoacusis	96	34.8
Psychiatric disorder	63	22.8
Brain lesion or other pathology	40	14.5
Epilepsy	12	4.4
Intoxication/pharmacology	63	22.8
None of the above	2	0.7
Total	276	100

### Treatment effects

In Table 3 the mean treatment effects per etiology and pharmacological class are displayed, as well as the number of patients with a complete response. A mean close to zero indicates no or little effect of the treatment, and a mean close to two indicates that the MH disappeared completely or almost completely.

**Table 3:** **Mean effect of (pharmacological) treatment on musical hallucinations (0 = no effect, 1 = clinically meaningful, 2 = complete disappearance)**.

**Main etiological factor**	**Treatment**	**Mean**	**Complete response *N* (%)**	***N***
Hypoacusis	Antipsychotic	0.52	6 (19%)	31
	Antiepileptic	0.89	6 (22%)	27
	Antidepressant	1	6 (32%)	19
	Acetylcholinesterase inhibitor	1.71	5 (71%)	7
	Total response of all patients	**1.19**	**24 (32%)**	**76**
Psychiatric disorder	Antipsychotic	0.75	10 (28%)	36
	Antiepileptic	1.18	4 (36%)	11
	Antidepressant	1, 18	10 (45%)	22
	Benzodiazepines	1.00	2 (40%)	5
Psychosis	Antipsychotics	1.00	6 (40%)	15
	Total	1.26	8 (42%)	19
Depression/Anxiety	Antidepressant	1.22	9 (50%)	18
	Antipsychotic	0.67	5 (21%)	24
	Total	1.32	14 (36%)	39
	Total response of all patients	**1.25**	**31 (49%)**	**63**
Brain lesion or other pathology	Total response of all patients	**1.66**	**28 (70%)**	**40**
Intoxication/pharmacology	Antipsychotic	0		2
	Antiepileptic	2.0	1	1
	Antidepressant	0		1
	Total response of all patients	**1.94**	**61 (97%)**	**63**

#### Hypoacusis

In the hypoacusis group, 80 out of 96 patients either received treatment or improved spontaneously, with a mean effect of 1.19. Twenty-two patients desired no treatment (in two patients after trying a treatment first) since they generally found that they could manage without. Although impaired hearing was considered the main etiological factor, the group had an average of 1.54 etiological factors. Eleven patients showed spontaneous improvement, often after reassurance. Behavioral interventions that were attempted included improving sleep or lying on one ear at night (which, incidentally, was not helpful during the day) (Keshavan et al., 1992). Several patients report that they were bothered less by their MH when they listened to the radio (Mahendran, 2007; Teunisse and Olde Rikkert, 2012), drummed along with their fingers (Raghuram et al., 1980), wore headphones (Keshavan et al., 1988), sang along (Berrios, 1990), listened to music at bedtime (Coebergh et al., 2009), rubbed the hair near their ears (Gilchrist and Kalucy, 1983), or used a tinnitus mask (i.e., a hearing device that delivers music-like tones) (Abdel-Aziz and Pomeroy, 2014).

Six patients had some response, and six a complete response to interventions directed at improving their hearing; e.g., (optimizing) hearing aids (e.g., Feehan and Birchwood, 1993; Tuerlings et al., 2009), removing ear wax (Terao and Matsunaga, 1999; Tuerlings et al., 2009), and otosclerosis surgery [which in one case replaced MH with tinnitus (Mocellin et al., 2008)]. MH occurred only when taking hearing aid out at night in one patient (Serrador-García et al., 2012). It was generally not recorded whether changes in the use of a hearing aid made any difference but several reports mention briefly that no change occurred. Cochlear implantation can be a cause of MH but in two cases adaptation of the implant did not lead to any meaningful improvement (Auffarth and Kropp, 2009; Joe et al., 2015).

Among the 54 patients treated pharmacologically; on average 1.95 types of medication (range 1–11) were tried. The mean treatment effect of acetylcholinesterase inhibitors was 1.71 in seven patients (Shinosaki et al., 2003; Ukai et al., 2007; Strauss and Gertz, 2009; Serrador-García et al., 2012; Colon-Rivera and Oldham, 2014; Blom et al., 2015). Five other patients were treated with benzodiazepines or barbiturates and their mean response was 0.66. The mean treatment effect of antidepressants in 19 patients was 1.0, with six showing a complete response. The mean treatment effect of antipsychotics in 31 patients was 0.52, with six of them achieving a complete response. The mean treatment effect of antiepileptics in 27 patients was 0.89, with six achieving a complete response.

#### Psychiatric disorder

Among the group diagnosed with a psychiatric disorder (63 patients) many were treated pharmacologically (54 patients). The patients had a mean response to treatment of 1.25, and an average of 1.65 causes.

One patient benefited from adjustment of his hearing aid, even though psychosis was considered the trigger for his MH (Keshavan et al., 1992). One benefited from singing along (Miller and Crosby, 1979), one from listening to the radio (Baurier and Tuca, 1996), and one from CBT (Wilkinson and Schuller, 2004). Four patients improved spontaneously and two did not want any intervention even thought there were significantly bothered by their MH (Podoll et al., 1991; Keshavan et al., 1992). Treating the underlying psychiatric disorder with ECT was helpful for MH in five patients, but not in all (Wengel et al., 1989; Stephane and Hsu, 1996; Janakiraman et al., 2006) and sometimes the effect was temporary (Colon-Rivera and Oldham, 2014).

The pharmacological treatment response was best when an antidepressant was used (1.18) (with 10 of them showing a complete response), or when antiepileptics were used (1.18), with four patients responding completely. Antipsychotics yielded a mean treatment response of 0.75, and complete disappearance of the MH in 10 patients. Results tended to be better in patients diagnosed with schizophrenia spectrum disorder (1.00) than in those with mood disorders (0.67). The use of benzodiazepines led to complete disappearance in two patients, and to a mean treatment response of 1.0 (in five patients).

#### Brain lesion and other pathology

In the brain lesion and other pathology group (40 patients) the mean number of causes was 1.4 (range 1–3). Sixteen cases of MH had a cerebrovascular cause, and in nine cases the cause was a tumor and others had pathology like multiple sclerosis and Parkinson's disease. When a tumor was present removal led to disappearance of MH (Penfield and Erickson, 1941; David et al., 1944; Scott, 1979). In all stroke cases, the hallucinations disappeared without any adjuvant treatment in days or months and up to a year [and in one case only ever lasted 90 min (Cerrato et al., 2001)]. In 22 patients MH disappeared without intervention.

One patient with a previous head trauma, who had not responded to antiepileptics and antidepressants, was treated with rTMS after 1 year of experiencing MH. The MH disappeared after 2 weeks, only to reappear after 4 months; although by that time they were less intense, and only present in a quiet environment and before falling asleep (Cosentino et al., 2010).

All in all it is difficult to fully ascertain any medication effects in the brain injury group. Thirteen patients were treated pharmacologically, but in one case of delirium (Rentrop et al., 2009) and in two cases of brain tumor patients the natural history probably led to improvement (Evers et al., 2002), rather than the use of medication (i.e., olanzapine and pregabalin, respectively). Four patients showed a complete response, and three no response to antiepileptics; three patients were treated with antidepressants, and four with antipsychotics, which are all very small numbers to establish a mean treatment response. Twenty-two improved spontaneously and six did not want any treatment.

#### Epilepsy

The rather small epilepsy group, which comprised of 12 patients, had a mean treatment response of 1.66. The mean number of medication used was 1.08 (range 1–4) in eight patients. MH tended to respond well to antiepileptics or surgery for the underlying cause (Rennie, 1964; Wieser, 1980; Roberts et al., 2001). Four cases of MH resolved spontaneously and were ictal or post-ictal in nature, and some of these patients were on antiepileptics already that was not changed or just started after the seizure but the MH were in all likelihood post-ictal.

#### Intoxication/pharmacology

The intoxication/pharmacology group comprised of 63 patients. The mean treatment result was a robust 1.94 when patients stopped their medication or switched to another type of medication. When the above-mentioned case series of 44 patients with alcoholic hallucinosis (Scott, 1975) was excluded, the mean treatment response was 1.79. The mean number of causes in the intoxication/pharmacology group was 1.4.

They include benzodiazepines [lormetazepam (Ginsberg, 2003), temazepam (Fisman, 1991), lorazepam (Curtin and Remund, 2002), and triazolam (Nevins, 1991)], opioids [morphine (Davies and Quinn, 2005), tramadol (Keeley et al., 2000) and oxycodone (Moore, 2003)], tricyclic antidepressants [imipramine (Terao, 1995), clomipramine (Vallada and Gentil, 1991) and mirtazapine (Padala et al., 2010)], anticholinergic medication [biperiden (Gertz et al., 1996)], alcohol (Scott, 1975), an N-methyl-D-aspartate antagonist [amantadine (Gondim et al., 2010)], a dopamine agonist [bromocriptine (Kobayashi et al., 2004)], salicylates (Allen, 2008), dipyridamole(Tomar and Cheung, 2007), propranolol (Fernandez and Crowther, 1998), voriconazole (Agrawal and Sherman, 2004), pentoxifylline (Gilbert, 1993) and steroids (Kanemura et al., 2010).

Only six patients in this group were treated with adjuvant medication. This had no effect, except in one patient who suffered from depression, white matter changes and a hearing impairment. Biperiden induced the MH and they responded to carbamazepine (Gertz et al., 1996). One patient benefited from listening to the radio (Fernandez and Crowther, 1998).

In 42 cases the musical hallucinations decreased or disappeared without any treatment—see Table 4. As indicated above, most of them stemmed from the brain lesion and other pathology group.

**Table 4:** **Decrease or disappearance of musical hallucinations without any intervention**.

Etiological factor	*N*
Hearing impairment	11
Psychiatric disorder	4
Brain lesion and other pathology	22
Epilepsy	4
None	1
Total	42

## Discussion

Musical hallucinations, like actual musical sounds, are associated with activity in an extensive network of interconnected brain areas. This network comprises auditory areas, visual areas, basal ganglia, brainstem, pons, tegmentum, cerebellum, hippocampi, amygdala, motor cortex, the peripheral auditory system, and possibly many other areas (Sacks and Blom, 2012). Even though there is not a single unique pathophysiological pathway within the network that can be held responsible for the mediation of MH, there is often an etiological factor that would seem to act as a necessary and sufficient condition for it to become activated. We hypothesized that the key to successful treatment of MH might lie in identifying that etiological factor. Based on our analysis of the treatment results of 276 patients with MH as described in the literature, the following can be said about this.

In the hypoacusis group—with patients suffering from idiopathic MH—frequently no treatment is required except for explanation, behavioral interventions, and improving auditory function. And yet pharmacological therapy can also be successful, with many different medications. The mean treatment response rate in this group is highest—although admittedly modest—with acetylcholinesterase inhibitors, followed by antiepileptics and antidepressants, and lowest with antipsychotics (which however can still be useful). Symptomatic MH, on the other hand, are more likely to respond to pharmacological treatment. In the group diagnosed with a psychiatric disorder, the type of disorder provides the most important clue to choice of treatment. Thus, treating MH experienced in the context of a depression with the aid of an antidepressant is more effective than with an antipsychotic, whereas MH experienced in the context of a schizophrenia spectrum disorder tend to respond more often to antipsychotics. Patients in the brain-injury and epilepsy groups can best be treated with antiepileptics, although in these groups the natural course of the MH is often benign, especially after a stroke. When intoxication/pharmacology is the main etiological factor, it is important to stop the medication that is held responsible for their mediation, and if necessary to replace it with a substance from a different pharmacological class.

As an aside, it is worth mentioning that in the intoxication/pharmacology group many causative medications play a role that have an opposite effect to medications used for treatment. Dopamine agonists (causative) and antipsychotics (treatment) work on the same dopaminergic receptors. Anticholinergics and tricyclics (causative) and acetylcholinesterase inhibitors (treatment) act on the same cholinergic neurotransmitters. Benzodiazepines, which act on the gamma-amino-butyric-acid (GABA) system, can at once be causative and curative. This suggests that all the neurotransmitter systems and receptors involved can play an a priori unpredictable role in the mediation *and* treatment of MH, and that proper clinical and pharmacological assessment is always necessary to establish the course of action that is most likely to yield favorable results.

### Limitations

This review has several limitations. Firstly, there would seem to be a positive publication bias for successful treatments. For example, salicylates (Allen, 2008) and propranolol (Fernandez and Crowther, 1998), which in some studies are associated with the mediation of MH, are used by many of our patients in clinical practice without causing this particular side effect. Moreover, we have attempted to treat some of our patients by withdrawing these medications, and no resolution of the MH was ever noted. None of the pharmacological treatments (or withdrawal) were ever placebo controlled. There may also be a positive publication bias for pharmacological treatments *per se*. Many of our patients, after proper explanation, prefer not to try any medication, whereas others try one type of medication but refrain from trying another type. In our experience some patients are content with explanation and practical advice, which is also described by others (Shapiro et al., 1991; Feehan and Birchwood, 1993; Bhatt and de Carpentier, 2012; Johns and Zuromskis, 2012; Teunisse and Olde Rikkert, 2012). However, the fact that patients show themselves content without any pharmacological intervention does not mean that no treatment should be offered (Podoll et al., 1991; Keshavan et al., 1992).

A second limitation of this review is that its sample size is relatively small (*N* = 276), especially when subdividing the group in etiological subgroups. A previous review included 132 patients (Evers and Ellger, 2004), and yet another one did not state the total number of patients included but instead based its conclusions on 55 relevant papers (Cope and Baguley, 2009). Our sample size is nevertheless the largest analyzed so far. With the small numbers involved it cannot be ruled out that the variations are due to chance since statistical significance of differential treatment responses in every etiological subgroup would often not have been reached.

Thirdly, we may have underestimated the effects of antipsychotics in the hypoacusis group by not including the majority of patients described by Warner and Aziz (2005). As noted above, we decided to count this group as a single patient because individual patient data were lacking in the original report. However, the mean response rate to antipsychotics in this study is much higher than in others. Moreover, by lumping various classes of antidepressants into a single group, we may have missed the opportunity to identify possible group differences, especially since tricyclics are implicated in various studies as possible causes of MH. The same holds true for the various groups of antipsychotics and antiepileptics that work on very different ion channels and neurotransmitters.

If patients have tried multiple drugs in one class but did respond to another one we scored this as a response and therefore overestimated the effect of individual drugs; some patients tried up to 11 medications (Colon-Rivera and Oldham, 2014).

The interpretation of the intoxication/pharmacology literature is influenced by inclusion of 44 patients with alcoholic musical hallucinosis out of a total of 63 patients (Scott, 1975). Removing them from the analysis did not alter the finding that withdrawing the presumed offending medication is highly effective.

### Conclusions and recommendations

Musical hallucinations are best treated by directing our intervention at the etiological mechanism responsible for their mediation. Based on the findings presented above and on our clinical experience with MH, our advice would be to chart the (possibly multiple) underlying causes with the aid of a history; extensively exploring temporal relationships between MH and medication, trauma, drug (ab-)use, seizures, and use of hearing aid devices. Furthermore, neurological assessment, psychiatric assessment an ear, nose, and throat (ENT) assessment are recommended, and imaging with an MRI head. Not all patients need treatment, as MH may be self-limiting, especially when occurring after brain lesions. If associated distress is low, reassurance may be enough. When treatment is indicated, we suggest that non-pharmacological interventions should be attempted first. This may involve interventions ranging from behavioral modifications, increasing social interactions and CBT. ENT interventions and withdrawing possible causative medication should be attempted due to their low risk of side effects. When idiopathic MH significantly affects the patient's quality of life, it is worthwhile to target any particularly troublesome symptom, i.e., anxiety with a selective serotonin reuptake inhibitor (SSRI), a secondary delusion with antipsychotics, and sleep disturbances with hypnotics. In the hypoacusis group, pharmacological treatment can first perhaps best be attempted with acetylcholinesterase inhibitors. Although there currently is only limited evidence for the effectiveness of this medication group, in our clinical experience they can help significantly, while side effects are relatively mild as compared to antipsychotic and antiepileptic medication.

Antidepressants and antiepileptics have a more favorable response rate and less side effects than antipsychotics; considering most patients are elderly. The literature documents several examples of beneficial effects using carbamazepine, often a low doses.

Targeting treatment to the underlying psychiatric disorder can clearly be helpful; MH in those with mood disorders respond less well to antipsychotics than in those with a psychotic disorder. ECT can clearly be helpful in patients with severe depression and MH (Wengel et al., 1989; Stephane and Hsu, 1996; Janakiraman et al., 2006), albeit sometimes relief of MH is only brief (Colon-Rivera and Oldham, 2014).

Further exploration of rTMS could be worthwhile after the successful treatment in a very treatment resistant case (Cosentino et al., 2010).

Finally, we would like to make recommendations for further research. There ought to be a clear documentation of the volume and frequency of the MH, degree of control over the MH, quality of the MH (as well as its relation to tinnitus), and the influence of MH on daily life, before and after the start of treatment. Documenting what is taken as a successful treatment response (i.e., less meaningful, less frequent/intrusive/loud, more control, complete disappearance, improved sleep) is also essential. It should be considered to try placebo-controlled treatment (and withdrawal of presumed offending medication). Larger sized treatment trials should be able to elucidate whether the differential treatment response to different (classes) of medication is real, as suggested by this review. This should allow for a more effective and accurate assessment of the effects of treatment in the future. Perhaps, analogous to recent initiatives to create international databases for rare diseases, a database for cases of MH should be considered, with a focus on diagnostic findings, natural course, and treatment results.

## Author contributions

JC designed the manuscript, acquired data, drafted the manuscript, gave final approval of the version to be published, and agreed to be accountable for all aspects of the work in ensuring that questions related to the accuracy or integrity of any part of the work are appropriately investigated and resolved. RL contributed substantially to the design of the manuscript, acquired data, revised the manuscript, gave final approval of the version to be published, and agreed to be accountable for all aspects of the work in ensuring that questions related to the accuracy or integrity of any part of the work are appropriately investigated and resolved. RB acquired data, helped to draft the manuscript, revised the manuscript, gave final approval of the version to be published, and agreed to be accountable for all aspects of the work in ensuring that questions related to the accuracy or integrity of any part of the work are appropriately investigated and resolved. IS contributed substantially to the design of the manuscript, revised the manuscript, gave final approval of the version to be published, and agreed to be accountable for all aspects of the work in ensuring that questions related to the accuracy or integrity of any part of the work are appropriately investigated and resolved. JB contributed substantially to the design of the manuscript, revised the manuscript, gave final approval of the version to be published, and agreed to be accountable for all aspects of the work in ensuring that questions related to the accuracy or integrity of any part of the work are appropriately investigated and resolved.

### Conflict of interest statement

The authors declare that the research was conducted in the absence of any commercial or financial relationships that could be construed as a potential conflict of interest.
